# Crystal structure of 4-methyl-7-prop­oxy-2*H*-chromen-2-one

**DOI:** 10.1107/S1600536814023678

**Published:** 2014-10-31

**Authors:** Samuel Estrada-Soto, Amanda Sánchez-Recillas, Gabriel Navarrete-Vázquez, Hugo Tlahuext

**Affiliations:** a Facultad de Farmacia, Universidad Autónoma del Estado de Morelos, Av. Universidad 1001 Col. Chamilpa CP 62100, Cuernavaca Mor., México; b Centro de Investigaciones Químicas, Universidad Autónoma del Estado de Morelos, Av. Universidad 1001 Col., Chamilpa, CP 62100, Cuernavaca Mor., México

**Keywords:** crystal structure, coumarin derivative, offset π–π inter­actions, C—H⋯O inter­actions

## Abstract

The asymmetric unit of the title compound, C_13_H_14_O_3_, contains two independent mol­ecules, *A* and *B*, that are inter­connected through an offset π–π inter­action [inter-centroid separation = 3.6087 (4) Å]. The fused benzene and pyran-2-one rings in each mol­ecule are essentially coplanar, having dihedral angles of 1.22 (12) and 1.57 (12)° for mol­ecules *A* and *B*, respectively. Similarly, the coumarin ring system and the 7-prop­oxy substituent are close to being coplanar [C—C—O—C torsion angles = 2.9 (2) and 1.4 (2)° for mol­ecules *A* and *B*, respectively]. In the crystal, the mol­ecules are connected by C—H⋯O hydrogen bonds, forming supra­molecular tapes along [100] that are linked into a three-dimensional network by C—H⋯π inter­actions, as well as by the aforementioned π–π inter­actions.

## Abstract 2

The asymmetric unit of the title compound contains two independent mol­ecules that are inter­connected through an offset π–π inter­action. The fused benzene and pyran-2-one rings in each mol­ecule are essentially coplanar. Similarly, the coumarin ring system and the 7-prop­oxy substituent are close to being coplanar.

## Chemical context   

Coumarin (2*H*-1-benzo­pyran-2-one) is a plant-derived natural product known for its pharmacological properties such as anti-inflammatory, anti­coagulant, anti­bacterial, anti­fungal, anti­viral, anti­cancer, anti­hypertensive, anti­tubercular, anti­convulsant, anti-adipogenic, anti­hyperglycemic, anti-oxidant and neuroprotective properties. Dietary exposure to benzopyrones is significant as these compounds are found in vegetables, fruits, seeds, nuts, coffee, tea and wine (Venugopala *et al.*, 2013 ▶). In order to assist our knowledge about the stereoelectronic requirements from these kinds of mol­ecules to show anti-asthmatic or tracheal relaxant actions, we have synthesized (Sánchez-Recillas *et al.*, 2014 ▶) and determined the crystal structure of the title compound, (I). A related structure, 3-acetyl­coumarin, has been reported on by Munshi *et al.* (2004 ▶).
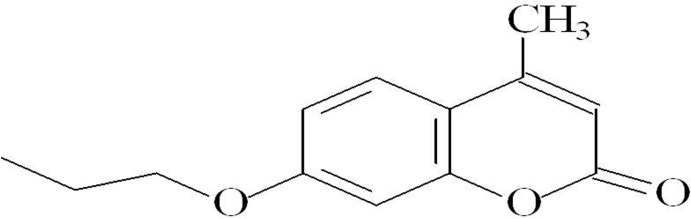


## Structural commentary   

The asymmetric unit of (I) contains two independent mol­ecules (*A* and *B*). Bond lengths between equivalent non-H atoms of each mol­ecule are similar, with differences less than 3 s.u.

The fused aryl and pyran-2-one rings in each mol­ecule are individually planar (r.m.s. deviations < 0.0064 for aryl rings and < 0.0141 Å for pyran-2-one rings) and form single planar units [r.m.s. deviation = 0.0159 Å, dihedral angle between the two six-membered rings of 1.22 (12)° in mol­ecule *A*; r.m.s. deviation = 0.0192 Å, dihedral angle between the two six-membered rings of 1.57 (12)° for *B*].

The torsion angles between the coumarins ring systems and the 7-prop­oxy substituents in *A* (C7—C6—O3—C10) and *B* (C20—C19—O6—C23) are 2.9 (2) and 1.4 (2)°, respectively. The two independent mol­ecules are inter­connected through an offset π–π inter­action, with a distance between the centroids of the C4–C9 and C17–C22 benzene rings of 3.6087 (4) Å.

## Supra­molecular features   

**Figure 2:**
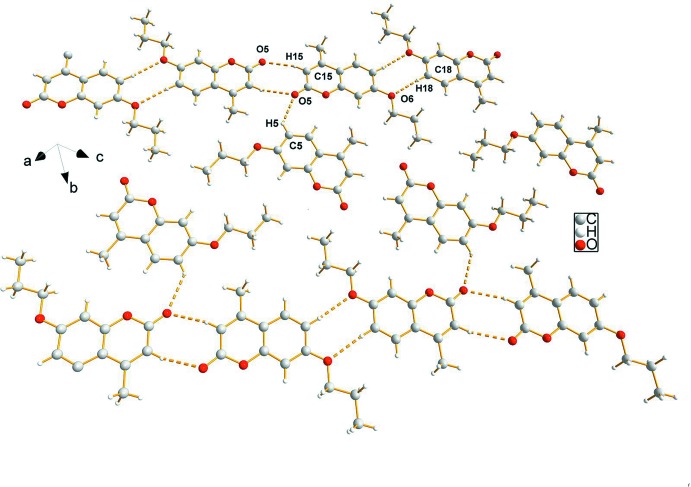
View of the supra­molecular tape structure along [100] sustained by C—H⋯O hydrogen bonds (dashed lines).

**Figure 3:**
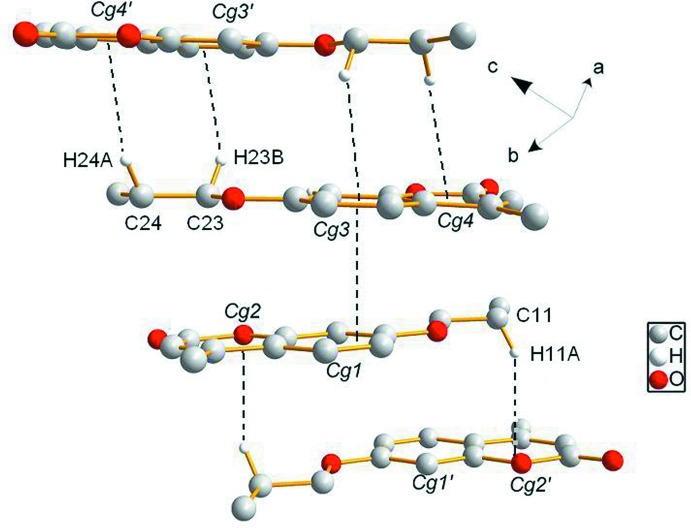
View of the C—H⋯π hydrogen bonds (dashed lines) between neighbouring tapes. *Cg*2′, *Cg*3′ [symmetry code: (′) −*x* + 1, −*y* + 2, −*z*] and *Cg*4′ [symmetry code: (′) −*x* + 1, −*y* + 1, −*z* + 1].

The packing is mainly through C—H⋯O and C—H⋯π hydrogen bonding (Table 1 ▶) as well as the π–π inter­action mentioned above. Three *B* mol­ecules are connected through two pairs of C—H⋯O hydrogen bonds, generating two centrosymmetric 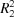
(8) graph sets; the first involving atoms (⋯H15–C15–C14–O5⋯)_2_ and the second involving atoms (⋯H18–C18–C19–O6⋯)_2._ The 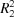
(8) motifs are connected with *A* mol­ecules through C—H⋯O contacts, generating a tape-like structure along [100] (Fig. 2 ▶). Additional, C—H⋯π inter­actions provide the links between neighboring tapes, resulting in a three-dimensional network (Fig. 3 ▶).

## Synthesis and crystallization   

The title compound was prepared by *SN*2 reaction between 7-hy­droxy-4-methyl-2*H*-chromen-2-one and *n*-propyl bromide. 7-Hy­droxy-4-methyl-2*H*-chromen-2-one (0.4 g, 2.27 mmol) and potassium carbonate (1.28 g, 9.30 mmol, 4.1 equiv) were dissolved in acetone (2.0 ml) and kept at room temperature. After 20 minutes, *n*-propyl­bromide (0.641 ml, 7.03 mmol) was added drop wise and the reaction mixture was heated to reflux (313 K) and monitored by TLC. After completion of the reaction (six days), the reaction mixture was filtered and the solid residue was washed off with cold water (10 ml). The total mother liquors were concentrated under reduced pressure and then poured into water and extracted with ethyl acetate (3 × 15 ml). The organic layer was dried over anhydrous Na_2_SO_4_ and evaporated under reduced pressure to give a white-coloured solid (m.p. 347.55–348.35 K). Single crystals were obtained from methanol. ^1^H NMR data (400 MHz; CDCl_3_: Me_4_Si) *d*: 1.02 (3*H*, *t*, CH_3_), 1.82 (2*H*, *m*, CH_2_), 2.36 (3*H*, *s*, CH_3_), 3.94 (2*H*, *t*, CH_2_—O), 6.08 (*s*, 1H, H-3), 6.76 (1*H*, *d* H, *J =*2.4 Hz, H-8), 6.82 (1*H*, *dd*, CH, *J =* 8.8 Hz, *J =* 2.4 Hz, H-6), 7.45 (1*H*, *d*, CH, *J =* 8.8 Hz, H-5).

### Refinement   

Crystal data, data collection and structure refinement details are summarized in Table 2 ▶. H atoms were positioned geometrically and constrained using the riding-model approximation [C—H_ar­yl_ = 0.95 Å, *U*_iso_(H_ar­yl_) = 1.2 *U*_eq_(C); C—H_methyl­ene_ = 0.99 Å, *U*_iso_(H_methyl­ene_) = 1.2 *U*_eq_(C); C—H_meth­yl_ = 0.98 Å, *U*_iso_(H_methy_l) = 1.5 *U*_eq_(C)].

## Supplementary Material

Crystal structure: contains datablock(s) I, New_Global_Publ_Block. DOI: 10.1107/S1600536814023678/tk5346sup1.cif

Structure factors: contains datablock(s) I. DOI: 10.1107/S1600536814023678/tk5346Isup2.hkl

CCDC reference: 1031270

Additional supporting information:  crystallographic information; 3D view; checkCIF report

## Figures and Tables

**Figure 1:**
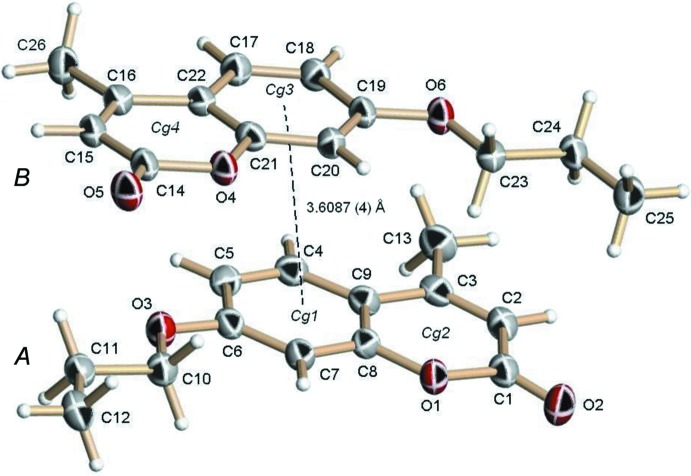
The mol­ecular structure of (I), showing the atom-labelling scheme and the offset π–π inter­action. Displacement ellipsoids are drawn at the 50% probability level and H atoms are shown as small spheres of arbitrary radius. The dashed line indicates the inter­action between the benzene ring centroids *Cg*1 (C4—C9) and *Cg2* (C17—C22).

**Table 1:** Hydrogen-bond geometry (, ) *Cg*2, *Cg*3 and *Cg*4 are the centroids of the O1/C1C3/C8/C9, C17C22 and O4/C14C16/C21/C22 rings, respectively.

*D*H*A*	*D*H	H*A*	*D* *A*	*D*H*A*
C5H5O5^i^	0.95	2.57	3.296(2)	133
C15H15O5^ii^	0.95	2.49	3.422(2)	165
C18H18O6^iii^	0.95	2.45	3.402(2)	175
C11H11*A* *Cg*2^iv^	0.99	2.76	3.6087(4)	140
C24H24*A* *Cg*4^v^	0.99	2.81	3.613(2)	139
C23H23*B* *Cg*3^v^	0.99	2.75	3.614(2)	145

Symmetry codes: (i) 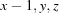; (ii) 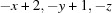; (iii) 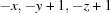; (iv) 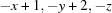; (v) 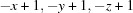.

**Table 2:** Experimental details

Crystal data	
Chemical formula	C_13_H_14_O_3_
*M*_r_	218.24
Crystal system, space group	Triclinic, *P* 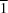
Temperature (K)	100
*a*, *b*, *c* ()	7.2418(9), 11.5459(14), 14.5301(17)
, , ()	69.014(2), 76.086(2), 84.124(2)
*V* (^3^)	1100.9(2)
*Z*	4
Radiation type	Mo *K*
(mm^1^)	0.09
Crystal size (mm)	0.25 0.25 0.21

Data collection	
Diffractometer	Bruker *SMART* *APEX* CCD area detector
Absorption correction	Multi-scan (*SADABS*; Sheldrick, 2003 ▶)
*T*_min_, *T*_max_	0.977, 0.981
No. of measured, independent and observed [*I* > 2(*I*)] reflections	9469, 3872, 3420
*R*_int_	0.030
(sin /)_max_ (^1^)	0.595

Refinement	
*R*[*F* ^2^ > 2(*F* ^2^)], *wR*(*F* ^2^), *S*	0.047, 0.122, 1.12
No. of reflections	3872
No. of parameters	293
H-atom treatment	H-atom parameters constrained
_max_, _min_ (e ^3^)	0.22, 0.25

Computer programs: *SMART* (Bruker, 2000 ▶), *SAINT-Plus* (Bruker, 2001 ▶), *DIAMOND* (Brandenburg, 1997 ▶), *SHELXTL-NT* (Sheldrick, 2008 ▶) and *publCIF* (Westrip, 2010 ▶).

## supplementary crystallographic information

### Crystal data

**Table:**

C_13_H_14_O_3_	*Z* = 4
*M**_r_* = 218.24	*F*(000) = 464
Triclinic, *P*1	*D*_x_ = 1.317 Mg m^−^^3^
Hall symbol: -P 1	Mo *K*α radiation, λ = 0.71073 Å
*a* = 7.2418 (9) Å	Cell parameters from 7718 reflections
*b* = 11.5459 (14) Å	θ = 2.8–28.2°
*c* = 14.5301 (17) Å	µ = 0.09 mm^−^^1^
α = 69.014 (2)°	*T* = 100 K
β = 76.086 (2)°	Block, colourless
γ = 84.124 (2)°	0.25 × 0.25 × 0.21 mm
*V* = 1100.9 (2) Å^3^	

### Data collection

**Table:**

Bruker SMART APEX CCD area-detector diffractometer	3872 independent reflections
Radiation source: fine-focus sealed tube	3420 reflections with *I* > 2σ(*I*)
Graphite monochromator	*R*_int_ = 0.030
Detector resolution: 8.3 pixels mm^-1^	θ_max_ = 25.0°, θ_min_ = 1.5°
φ and ω scans	*h* = −8→8
Absorption correction: multi-scan (*SADABS*; Sheldrick, 2003)	*k* = −13→13
*T*_min_ = 0.977, *T*_max_ = 0.981	*l* = −17→17
9469 measured reflections	

### Refinement

**Table:**

Refinement on *F*^2^	Primary atom site location: structure-invariant direct methods
Least-squares matrix: full	Secondary atom site location: difference Fourier map
*R*[*F*^2^ > 2σ(*F*^2^)] = 0.047	Hydrogen site location: inferred from neighbouring sites
*wR*(*F*^2^) = 0.122	H-atom parameters constrained
*S* = 1.12	*w* = 1/[σ^2^(*F*_o_^2^) + (0.0454*P*)^2^ + 0.5831*P*] where *P* = (*F*_o_^2^ + 2*F*_c_^2^)/3
3872 reflections	(Δ/σ)_max_ = 0.001
293 parameters	Δρ_max_ = 0.22 e Å^−^^3^
0 restraints	Δρ_min_ = −0.25 e Å^−^^3^

### Special details

**Table:**

Geometry. All e.s.d.'s (except the e.s.d. in the dihedral angle between two l.s. planes) are estimated using the full covariance matrix. The cell e.s.d.'s are taken into account individually in the estimation of e.s.d.'s in distances, angles and torsion angles; correlations between e.s.d.'s in cell parameters are only used when they are defined by crystal symmetry. An approximate (isotropic) treatment of cell e.s.d.'s is used for estimating e.s.d.'s involving l.s. planes.
Refinement. Refinement of *F*^2^ against ALL reflections. The weighted *R*-factor *wR* and goodness of fit *S* are based on *F*^2^, conventional *R*-factors *R* are based on *F*, with *F* set to zero for negative *F*^2^. The threshold expression of *F*^2^ > σ(*F*^2^) is used only for calculating *R*-factors(gt) *etc*. and is not relevant to the choice of reflections for refinement. *R*-factors based on *F*^2^ are statistically about twice as large as those based on *F*, and *R*- factors based on ALL data will be even larger.

### Fractional atomic coordinates and isotropic or equivalent isotropic displacement parameters (Å^2^)

**Table:**

	*x*	*y*	*z*	*U*_iso_*/*U*_eq_	
C1	0.2312 (2)	1.02587 (16)	0.34492 (13)	0.0272 (4)	
C2	0.0602 (2)	0.95556 (16)	0.37805 (13)	0.0274 (4)	
H2	−0.0354	0.9666	0.4320	0.033*	
C3	0.0295 (2)	0.87428 (16)	0.33576 (13)	0.0269 (4)	
C4	0.1609 (3)	0.77466 (16)	0.20345 (13)	0.0269 (4)	
H4	0.0473	0.7297	0.2212	0.032*	
C5	0.3071 (2)	0.75950 (16)	0.12847 (13)	0.0265 (4)	
H5	0.2937	0.7048	0.0948	0.032*	
C6	0.4758 (2)	0.82451 (15)	0.10153 (12)	0.0253 (4)	
C7	0.4952 (2)	0.90553 (15)	0.14979 (12)	0.0248 (4)	
H7	0.6091	0.9504	0.1317	0.030*	
C8	0.3444 (2)	0.91946 (15)	0.22501 (12)	0.0235 (4)	
C9	0.1753 (2)	0.85505 (15)	0.25466 (12)	0.0240 (4)	
C10	0.7927 (2)	0.86097 (16)	−0.00035 (13)	0.0281 (4)	
H10A	0.8528	0.8348	0.0586	0.034*	
H10B	0.7742	0.9522	−0.0230	0.034*	
C11	0.9176 (3)	0.82336 (17)	−0.08476 (13)	0.0295 (4)	
H11A	0.8568	0.8502	−0.1434	0.035*	
H11B	0.9329	0.7319	−0.0620	0.035*	
C12	1.1119 (3)	0.88242 (19)	−0.11598 (14)	0.0347 (4)	
H12A	1.0966	0.9729	−0.1394	0.052*	
H12B	1.1919	0.8573	−0.1707	0.052*	
H12C	1.1724	0.8550	−0.0580	0.052*	
C13	−0.1524 (3)	0.80431 (18)	0.37212 (14)	0.0346 (4)	
H13A	−0.2305	0.8233	0.4303	0.052*	
H13B	−0.1237	0.7151	0.3921	0.052*	
H13C	−0.2219	0.8288	0.3177	0.052*	
C14	0.8545 (2)	0.54134 (16)	0.14392 (12)	0.0250 (4)	
C15	0.7477 (2)	0.46641 (15)	0.11512 (12)	0.0253 (4)	
H15	0.8064	0.4400	0.0599	0.030*	
C16	0.5668 (2)	0.43214 (15)	0.16371 (12)	0.0252 (4)	
C17	0.2919 (2)	0.44277 (16)	0.30600 (13)	0.0255 (4)	
H17	0.2194	0.3891	0.2924	0.031*	
C18	0.2136 (2)	0.48829 (15)	0.38246 (13)	0.0254 (4)	
H18	0.0881	0.4665	0.4208	0.030*	
C19	0.3192 (2)	0.56707 (15)	0.40370 (12)	0.0232 (4)	
C20	0.5028 (2)	0.59811 (15)	0.34929 (12)	0.0230 (4)	
H20	0.5755	0.6507	0.3639	0.028*	
C21	0.5781 (2)	0.55004 (15)	0.27239 (12)	0.0224 (4)	
C22	0.4766 (2)	0.47361 (14)	0.24749 (12)	0.0223 (4)	
C23	0.3305 (2)	0.69312 (16)	0.50284 (13)	0.0250 (4)	
H23A	0.3647	0.7685	0.4426	0.030*	
H23B	0.4491	0.6526	0.5226	0.030*	
C24	0.2037 (3)	0.72763 (16)	0.58868 (13)	0.0280 (4)	
H24A	0.1709	0.6519	0.6489	0.034*	
H24B	0.0841	0.7664	0.5690	0.034*	
C25	0.3047 (3)	0.81783 (18)	0.61425 (14)	0.0357 (4)	
H25A	0.4244	0.7799	0.6322	0.054*	
H25B	0.2232	0.8373	0.6714	0.054*	
H25C	0.3315	0.8943	0.5555	0.054*	
C26	0.4575 (3)	0.35469 (17)	0.13210 (14)	0.0324 (4)	
H26A	0.5353	0.3388	0.0725	0.049*	
H26B	0.3402	0.3991	0.1158	0.049*	
H26C	0.4255	0.2757	0.1874	0.049*	
O1	0.37241 (17)	1.00201 (11)	0.26973 (9)	0.0264 (3)	
O2	0.26525 (19)	1.10374 (12)	0.37645 (10)	0.0359 (3)	
O3	0.61273 (17)	0.80147 (11)	0.02699 (9)	0.0291 (3)	
O4	0.76161 (16)	0.58432 (11)	0.22148 (8)	0.0243 (3)	
O5	1.01862 (17)	0.57246 (12)	0.10667 (9)	0.0315 (3)	
O6	0.22868 (16)	0.60943 (11)	0.48005 (9)	0.0266 (3)	

### Atomic displacement parameters (Å^2^)

**Table:**

	*U*^11^	*U*^22^	*U*^33^	*U*^12^	*U*^13^	*U*^23^
C1	0.0281 (9)	0.0300 (9)	0.0229 (8)	0.0023 (7)	−0.0038 (7)	−0.0103 (7)
C2	0.0273 (9)	0.0319 (9)	0.0217 (8)	0.0009 (7)	−0.0034 (7)	−0.0093 (7)
C3	0.0267 (9)	0.0288 (9)	0.0214 (8)	0.0006 (7)	−0.0061 (7)	−0.0039 (7)
C4	0.0294 (9)	0.0264 (9)	0.0245 (9)	−0.0029 (7)	−0.0089 (7)	−0.0058 (7)
C5	0.0315 (9)	0.0253 (8)	0.0250 (9)	−0.0007 (7)	−0.0107 (7)	−0.0086 (7)
C6	0.0286 (9)	0.0270 (9)	0.0192 (8)	0.0025 (7)	−0.0052 (7)	−0.0074 (7)
C7	0.0263 (9)	0.0271 (9)	0.0211 (8)	−0.0010 (7)	−0.0046 (7)	−0.0088 (7)
C8	0.0286 (9)	0.0234 (8)	0.0203 (8)	0.0007 (7)	−0.0083 (7)	−0.0080 (7)
C9	0.0252 (8)	0.0248 (8)	0.0209 (8)	−0.0009 (7)	−0.0070 (7)	−0.0052 (7)
C10	0.0288 (9)	0.0300 (9)	0.0256 (9)	0.0003 (7)	−0.0048 (7)	−0.0108 (7)
C11	0.0329 (10)	0.0323 (9)	0.0222 (9)	0.0026 (7)	−0.0047 (7)	−0.0100 (7)
C12	0.0331 (10)	0.0464 (11)	0.0256 (9)	0.0030 (8)	−0.0063 (8)	−0.0147 (8)
C13	0.0295 (10)	0.0400 (11)	0.0313 (10)	−0.0058 (8)	−0.0032 (8)	−0.0095 (8)
C14	0.0265 (9)	0.0283 (9)	0.0181 (8)	0.0021 (7)	−0.0005 (7)	−0.0089 (7)
C15	0.0284 (9)	0.0276 (9)	0.0201 (8)	0.0010 (7)	−0.0023 (7)	−0.0106 (7)
C16	0.0285 (9)	0.0250 (8)	0.0210 (8)	0.0001 (7)	−0.0038 (7)	−0.0079 (7)
C17	0.0252 (9)	0.0266 (8)	0.0251 (9)	−0.0019 (7)	−0.0040 (7)	−0.0101 (7)
C18	0.0232 (8)	0.0270 (9)	0.0236 (8)	−0.0028 (7)	0.0002 (7)	−0.0088 (7)
C19	0.0242 (8)	0.0253 (8)	0.0180 (8)	0.0017 (6)	−0.0002 (6)	−0.0086 (6)
C20	0.0241 (8)	0.0239 (8)	0.0206 (8)	−0.0009 (7)	−0.0022 (7)	−0.0087 (7)
C21	0.0197 (8)	0.0237 (8)	0.0193 (8)	0.0001 (6)	−0.0006 (6)	−0.0047 (6)
C22	0.0234 (8)	0.0224 (8)	0.0201 (8)	0.0002 (6)	−0.0029 (7)	−0.0076 (7)
C23	0.0251 (8)	0.0279 (9)	0.0229 (8)	−0.0001 (7)	−0.0038 (7)	−0.0108 (7)
C24	0.0298 (9)	0.0329 (9)	0.0212 (8)	0.0052 (7)	−0.0038 (7)	−0.0120 (7)
C25	0.0441 (11)	0.0377 (10)	0.0306 (10)	0.0074 (9)	−0.0113 (8)	−0.0182 (8)
C26	0.0341 (10)	0.0368 (10)	0.0293 (10)	−0.0038 (8)	−0.0030 (8)	−0.0169 (8)
O1	0.0283 (6)	0.0305 (6)	0.0229 (6)	−0.0031 (5)	−0.0023 (5)	−0.0136 (5)
O2	0.0382 (7)	0.0401 (7)	0.0359 (7)	−0.0036 (6)	−0.0031 (6)	−0.0232 (6)
O3	0.0304 (7)	0.0333 (7)	0.0250 (6)	−0.0018 (5)	−0.0018 (5)	−0.0142 (5)
O4	0.0210 (6)	0.0302 (6)	0.0208 (6)	−0.0024 (5)	0.0024 (5)	−0.0118 (5)
O5	0.0252 (7)	0.0418 (7)	0.0267 (7)	−0.0050 (5)	0.0036 (5)	−0.0158 (6)
O6	0.0242 (6)	0.0336 (7)	0.0238 (6)	−0.0030 (5)	0.0018 (5)	−0.0158 (5)

### Geometric parameters (Å, º)

**Table:**

C1—O2	1.214 (2)	C14—O5	1.214 (2)
C1—O1	1.391 (2)	C14—O4	1.3916 (19)
C1—C2	1.437 (2)	C14—C15	1.440 (2)
C2—C3	1.353 (3)	C15—C16	1.353 (2)
C2—H2	0.9500	C15—H15	0.9500
C3—C9	1.447 (2)	C16—C22	1.452 (2)
C3—C13	1.498 (2)	C16—C26	1.501 (2)
C4—C5	1.372 (2)	C17—C18	1.373 (2)
C4—C9	1.406 (2)	C17—C22	1.405 (2)
C4—H4	0.9500	C17—H17	0.9500
C5—C6	1.399 (2)	C18—C19	1.402 (2)
C5—H5	0.9500	C18—H18	0.9500
C6—O3	1.365 (2)	C19—O6	1.3685 (19)
C6—C7	1.390 (2)	C19—C20	1.382 (2)
C7—C8	1.389 (2)	C20—C21	1.394 (2)
C7—H7	0.9500	C20—H20	0.9500
C8—O1	1.385 (2)	C21—O4	1.3769 (19)
C8—C9	1.394 (2)	C21—C22	1.392 (2)
C10—O3	1.438 (2)	C23—O6	1.442 (2)
C10—C11	1.510 (2)	C23—C24	1.513 (2)
C10—H10A	0.9900	C23—H23A	0.9900
C10—H10B	0.9900	C23—H23B	0.9900
C11—C12	1.523 (3)	C24—C25	1.524 (3)
C11—H11A	0.9900	C24—H24A	0.9900
C11—H11B	0.9900	C24—H24B	0.9900
C12—H12A	0.9800	C25—H25A	0.9800
C12—H12B	0.9800	C25—H25B	0.9800
C12—H12C	0.9800	C25—H25C	0.9800
C13—H13A	0.9800	C26—H26A	0.9800
C13—H13B	0.9800	C26—H26B	0.9800
C13—H13C	0.9800	C26—H26C	0.9800
			
O2—C1—O1	116.67 (16)	O4—C14—C15	117.51 (14)
O2—C1—C2	126.41 (16)	C16—C15—C14	122.54 (15)
O1—C1—C2	116.92 (15)	C16—C15—H15	118.7
C3—C2—C1	122.82 (16)	C14—C15—H15	118.7
C3—C2—H2	118.6	C15—C16—C22	118.65 (16)
C1—C2—H2	118.6	C15—C16—C26	121.73 (15)
C2—C3—C9	119.15 (16)	C22—C16—C26	119.61 (15)
C2—C3—C13	120.81 (16)	C18—C17—C22	121.56 (16)
C9—C3—C13	120.03 (16)	C18—C17—H17	119.2
C5—C4—C9	121.63 (16)	C22—C17—H17	119.2
C5—C4—H4	119.2	C17—C18—C19	119.83 (15)
C9—C4—H4	119.2	C17—C18—H18	120.1
C4—C5—C6	119.99 (16)	C19—C18—H18	120.1
C4—C5—H5	120.0	O6—C19—C20	123.65 (15)
C6—C5—H5	120.0	O6—C19—C18	115.75 (14)
O3—C6—C7	124.58 (16)	C20—C19—C18	120.60 (15)
O3—C6—C5	115.22 (15)	C19—C20—C21	118.08 (16)
C7—C6—C5	120.20 (16)	C19—C20—H20	121.0
C8—C7—C6	118.48 (16)	C21—C20—H20	121.0
C8—C7—H7	120.8	O4—C21—C22	121.81 (14)
C6—C7—H7	120.8	O4—C21—C20	115.06 (15)
O1—C8—C7	115.64 (15)	C22—C21—C20	123.13 (15)
O1—C8—C9	121.49 (15)	C21—C22—C17	116.77 (15)
C7—C8—C9	122.87 (16)	C21—C22—C16	118.50 (15)
C8—C9—C4	116.82 (16)	C17—C22—C16	124.73 (15)
C8—C9—C3	118.18 (15)	O6—C23—C24	108.36 (13)
C4—C9—C3	125.00 (16)	O6—C23—H23A	110.0
O3—C10—C11	107.78 (14)	C24—C23—H23A	110.0
O3—C10—H10A	110.2	O6—C23—H23B	110.0
C11—C10—H10A	110.2	C24—C23—H23B	110.0
O3—C10—H10B	110.2	H23A—C23—H23B	108.4
C11—C10—H10B	110.2	C23—C24—C25	110.15 (15)
H10A—C10—H10B	108.5	C23—C24—H24A	109.6
C10—C11—C12	110.13 (15)	C25—C24—H24A	109.6
C10—C11—H11A	109.6	C23—C24—H24B	109.6
C12—C11—H11A	109.6	C25—C24—H24B	109.6
C10—C11—H11B	109.6	H24A—C24—H24B	108.1
C12—C11—H11B	109.6	C24—C25—H25A	109.5
H11A—C11—H11B	108.1	C24—C25—H25B	109.5
C11—C12—H12A	109.5	H25A—C25—H25B	109.5
C11—C12—H12B	109.5	C24—C25—H25C	109.5
H12A—C12—H12B	109.5	H25A—C25—H25C	109.5
C11—C12—H12C	109.5	H25B—C25—H25C	109.5
H12A—C12—H12C	109.5	C16—C26—H26A	109.5
H12B—C12—H12C	109.5	C16—C26—H26B	109.5
C3—C13—H13A	109.5	H26A—C26—H26B	109.5
C3—C13—H13B	109.5	C16—C26—H26C	109.5
H13A—C13—H13B	109.5	H26A—C26—H26C	109.5
C3—C13—H13C	109.5	H26B—C26—H26C	109.5
H13A—C13—H13C	109.5	C8—O1—C1	121.32 (13)
H13B—C13—H13C	109.5	C6—O3—C10	117.68 (13)
O5—C14—O4	115.87 (15)	C21—O4—C14	120.86 (13)
O5—C14—C15	126.62 (15)	C19—O6—C23	117.62 (13)
			
O2—C1—C2—C3	176.86 (18)	O6—C19—C20—C21	−179.18 (14)
O1—C1—C2—C3	−3.6 (3)	C18—C19—C20—C21	0.7 (2)
C1—C2—C3—C9	1.0 (3)	C19—C20—C21—O4	−179.80 (14)
C1—C2—C3—C13	−179.20 (16)	C19—C20—C21—C22	0.6 (3)
C9—C4—C5—C6	0.2 (3)	O4—C21—C22—C17	178.73 (14)
C4—C5—C6—O3	179.21 (15)	C20—C21—C22—C17	−1.7 (2)
C4—C5—C6—C7	−0.7 (3)	O4—C21—C22—C16	−1.6 (2)
O3—C6—C7—C8	−179.56 (14)	C20—C21—C22—C16	177.96 (15)
C5—C6—C7—C8	0.3 (2)	C18—C17—C22—C21	1.5 (2)
C6—C7—C8—O1	−179.66 (14)	C18—C17—C22—C16	−178.13 (16)
C6—C7—C8—C9	0.5 (3)	C15—C16—C22—C21	1.9 (2)
O1—C8—C9—C4	179.26 (14)	C26—C16—C22—C21	−177.11 (15)
C7—C8—C9—C4	−0.9 (2)	C15—C16—C22—C17	−178.45 (16)
O1—C8—C9—C3	−1.4 (2)	C26—C16—C22—C17	2.6 (3)
C7—C8—C9—C3	178.37 (15)	O6—C23—C24—C25	−179.12 (14)
C5—C4—C9—C8	0.5 (2)	C7—C8—O1—C1	178.93 (14)
C5—C4—C9—C3	−178.70 (16)	C9—C8—O1—C1	−1.2 (2)
C2—C3—C9—C8	1.5 (2)	O2—C1—O1—C8	−176.75 (15)
C13—C3—C9—C8	−178.27 (16)	C2—C1—O1—C8	3.6 (2)
C2—C3—C9—C4	−179.23 (16)	C7—C6—O3—C10	2.9 (2)
C13—C3—C9—C4	1.0 (3)	C5—C6—O3—C10	−176.98 (14)
O3—C10—C11—C12	−179.21 (14)	C11—C10—O3—C6	−178.95 (13)
O5—C14—C15—C16	177.33 (17)	C22—C21—O4—C14	−1.3 (2)
O4—C14—C15—C16	−3.4 (2)	C20—C21—O4—C14	179.11 (14)
C14—C15—C16—C22	0.6 (3)	O5—C14—O4—C21	−176.94 (14)
C14—C15—C16—C26	179.60 (16)	C15—C14—O4—C21	3.7 (2)
C22—C17—C18—C19	−0.3 (3)	C20—C19—O6—C23	1.4 (2)
C17—C18—C19—O6	179.01 (14)	C18—C19—O6—C23	−178.55 (14)
C17—C18—C19—C20	−0.9 (3)	C24—C23—O6—C19	179.83 (13)

### Hydrogen-bond geometry (Å, º)

Cg2, Cg3 and Cg4 are the centroids of the O1/C1–C3/C8/C9, C17–C22 and O4/C14–C16/C21/C22 rings, respectively.

**Table:**

*D*—H···*A*	*D*—H	H···*A*	*D*···*A*	*D*—H···*A*
C5—H5···O5^i^	0.95	2.57	3.296 (2)	133
C15—H15···O5^ii^	0.95	2.49	3.422 (2)	165
C18—H18···O6^iii^	0.95	2.45	3.402 (2)	175
C11—H11*A*···*Cg*2^iv^	0.99	2.76	3.6087 (4)	140
C24—H24*A*···*Cg*4^v^	0.99	2.81	3.613 (2)	139
C23—H23*B*···*Cg*3^v^	0.99	2.75	3.614 (2)	145

Symmetry codes: (i) *x*−1, *y*, *z*; (ii) −*x*+2, −*y*+1, −*z*; (iii) −*x*, −*y*+1, −*z*+1; (iv) −*x*+1, −*y*+2, −*z*; (v) −*x*+1, −*y*+1, −*z*+1.

## References

[bb1] Brandenburg, K. (1997). *DIAMOND*. Crystal Impact GbR, Bonn, Germany.

[bb2] Bruker (2000). *SMART*. Bruker AXS Inc., Madison, Wisconsin, USA.

[bb3] Bruker (2001). *SAINT-Plus for Windows NT*. Bruker AXS Inc., Madison, Wisconsin, USA.

[bb4] Munshi, P., Venugopala, K. N., Jayashree, B. S. & Guru Row, T. N. (2004). *Cryst. Growth Des.* **4**, 1105–1107.

[bb5] Sánchez-Recillas, A., Navarrete-Vázquez, G., Hidalgo-Figueroa, S., Rios, M. Y., Ibarra-Barajas, M. & Estrada-Soto, S. (2014). *Eur. J. Med. Chem.* **77**, 400–408. 10.1016/j.ejmech.2014.03.029 24681028

[bb6] Sheldrick, G. M. (2003). *SADABS*. Bruker AXS Inc. Madison, Wisconsin, USA.

[bb7] Sheldrick, G. M. (2008). *Acta Cryst.* A**64**, 112–122. 10.1107/S0108767307043930 18156677

[bb8] Venugopala, K. N., Rashmi, V. & Odhav, B. (2013). *Biomed. Res. Int.* pp. 1–14. 10.1155/2013/963248 PMC3622347 23586066

[bb9] Westrip, S. P. (2010). *J. Appl. Cryst.* **43**, 920–925.

